# Diagnostic Classification of Schizophrenia Patients on the Basis of Regional Reward-Related fMRI Signal Patterns

**DOI:** 10.1371/journal.pone.0119089

**Published:** 2015-03-23

**Authors:** Stefan P. Koch, Claudia Hägele, John-Dylan Haynes, Andreas Heinz, Florian Schlagenhauf, Philipp Sterzer

**Affiliations:** 1 Department of Psychiatry and Psychotherapy, Campus Charité Mitte, Charité–Universitätsmedizin Berlin, Germany; 2 Bernstein Center for Computational Neuroscience Berlin, Charité–Universitätsmedizin Berlin, Germany; 3 Max Planck Institute for Human Cognitive and Brain Sciences, Leipzig, Germany; Indiana University, UNITED STATES

## Abstract

Functional neuroimaging has provided evidence for altered function of mesolimbic circuits implicated in reward processing, first and foremost the ventral striatum, in patients with schizophrenia. While such findings based on significant group differences in brain activations can provide important insights into the pathomechanisms of mental disorders, the use of neuroimaging results from standard univariate statistical analysis for individual diagnosis has proven difficult. In this proof of concept study, we tested whether the predictive accuracy for the diagnostic classification of schizophrenia patients vs. healthy controls could be improved using multivariate pattern analysis (MVPA) of regional functional magnetic resonance imaging (fMRI) activation patterns for the anticipation of monetary reward. With a searchlight MVPA approach using support vector machine classification, we found that the diagnostic category could be predicted from local activation patterns in frontal, temporal, occipital and midbrain regions, with a maximal cluster peak classification accuracy of 93% for the right pallidum. Region-of-interest based MVPA for the ventral striatum achieved a maximal cluster peak accuracy of 88%, whereas the classification accuracy on the basis of standard univariate analysis reached only 75%. Moreover, using support vector regression we could additionally predict the severity of negative symptoms from ventral striatal activation patterns. These results show that MVPA can be used to substantially increase the accuracy of diagnostic classification on the basis of task-related fMRI signal patterns in a regionally specific way.

## Introduction

Alterations in the neural processing of reward are a key finding in schizophrenia and have been proposed to be linked to dysfunctional dopaminergic neurotransmission in the mesolimbic reward system, first and foremost the central and ventral striatum [1–5]. Over the past decade, a number of functional magnetic resonance imaging (fMRI) studies have provided consistent evidence for reduced functional activation in the ventral striatum in response to reward-predicting stimuli in schizophrenia patients compared to controls [6–9]. This reduction in ventral striatal activation has been linked predominantly to the negative symptoms of schizophrenia [7,10]. In addition, reduced activation during reward processing in schizophrenia patients has also been observed in a number of other brain regions such as the amygdala, hippocampus, nucleus accumbens, prefrontal and insular cortex and parahippocampal gyrus [7,11–14]. While such findings based on significant group differences in fMRI signal have undoubtedly provided important insights into the pathomechanisms of schizophrenia, the use of such neuroimaging results from standard univariate statistical analysis for individual diagnosis has proven difficult, mostly because of large inter-individual variance in regional fMRI activations. An approach that can be used to overcome these difficulties is the use of multivariate pattern analysis (MVPA), which can dramatically increase the sensitivity of human brain imaging by accumulating information across multiple voxels of MRI signal, i.e., by taking into account the information contained in a distributed spatial pattern of brain activity rather than a single voxel or location [15,16]. A commonly applied implementation of MVPA is the use of a classification algorithm, e.g., support vector machine classification [17,18], that is trained to distinguish between two classes of data using pattern-based information. The accuracy of the trained classifier is then probed in independent test data. Such techniques have proven extremely useful not only for the decoding of brain states from patterns of brain imaging data on the individual-subject level but also for between-subject classification of brain imaging data in a number of psychiatric and neurological diseases (for reviews, see [19–22]). In recent years schizophrenia has been studied with MVPA using various neuroimaging variables such as resting state, diffusion tensor imaging and structural morphometry [23–28]. However, few studies have used MVPA to differentiate between schizophrenia patients and healthy controls on the basis of task-related fMRI signal patterns [29,30].

Here we asked whether MVPA could be used for the diagnostic classification of patients with schizophrenia vs. healthy controls on the basis of reward-related fMRI signal patterns obtained in a previous study [31]. In contrast to earlier studies that used MVPA for diagnostic classification [29,30], we were particularly interested in the regional specificity of MVPA-based classification, especially with respect to the above-mentioned brain regions that were implicated in altered reward processing in schizophrenia patients by earlier studies. Rather than using whole-brain activation patterns for classification, we employed a ‘searchlight’ approach [32,33] that can be used to assess classification accuracy for regional fMRI signal patterns across a whole fMRI scan volume [34,35]. Under this approach the searchlight is moved through the entire brain, and at each location, combines local information of voxels within a spherical volume across subjects. As the combined information of voxels within the sphere is projected to the center of the sphere at each location this approach eventually provides a whole-brain map of local information. Compared to other whole-brain approaches, searchlight MVPA offers some advantages such as the simplicity of implementation and the intuitive interpretation of the resulting maps similar to mass-univariate statistics. Moreover, searchlight MVPA circumvents the necessity for feature selection, which is a challenge for whole-brain MVPA due to high dimensionality. Finally, the searchlight approach preserves the regional specificity, thus allowing for a comparison of multivariate results with those obtained from mass-univariate methods. Because functional imaging of schizophrenia patients during a MID task has to date been exclusively analysed with mass-univariate statistics, we reasoned that the latter aspect is of particular relevance to benchmark MVPA against the standard univariate approach. We hypothesized that regionally specific classification accuracy would be highest for those brain regions whose reward-related activation has been previously shown to be altered in patients with schizophrenia, especially the ventral striatum [6–9,11–14,31,36]. In addition, we also asked whether MVPA of regional reward-related fMRI signal patterns could be used to predict the severity of clinical symptoms.

## Materials and Methods

### Participants

The study was approved by the local ethics committee, Charité–Universitätsmedizin, Berlin, Germany. Written informed consent was obtained from all participants. A total of 98 participants were included in the study: 54 healthy controls and 44 patients diagnosed with schizophrenia. Patients fulfilling DSM-IV and ICD-10 criteria for schizophrenia without having other psychiatric axis I disorders, current drug abuse or past history of drug dependence (SCID interview; [37]) were recruited at the Charité University Medical Centre's Department of Psychiatry and Psychotherapy. Psychopathological symptoms were assessed with the Positive and Negative Syndrome Scale (PANSS; [38]). Healthy participants in the control group showed no psychiatric axis I or II disorders (SCID) or any family history of psychiatric disorders and no substance abuse or dependence within the previous 6 months. Equal sample sizes in both groups were obtained by excluding datasets from the group with larger samples (healthy controls) based on a matching of age and gender criteria. Thus, the groups contained 44 schizophrenia patients (mean age: 34.2±9.8, range 19–57) and 44 healthy controls (mean age: 37.1±10.9, range 18–59), respectively. A two-sample t-test revealed no age differences between the two groups (t = 1.32, p = 0.19). There were 35 male controls (M:F ratio = 3.88) and 27 male patients (M:F ratio = 1.58). A Pearsons’s chi-square test revealed no differences between the two groups with respect to gender (chi = 3.49, p = 0.06). The medication status of the patients with schizophrenia consisted of 7 patients taking atypical antipsychotics, 21 conventional antipsychotics, and 16 not receiving any medication. All participants were right-handed, as assessed with the Edinburgh Handedness Inventory [39]. For a detailed description of the sample see Table 1.

**Table 1:** Demographic parameters and reaction times.

	**Healthy Controls**	**Schizophrenia**	**Study population**	**Between group differences**
Subjects [N]	44	44	88	
Males/Females [N]	35/9	27/17	62/26	X^2^ = 3.49, p = 0.06^a^
Age [years], mean (STD)	37.1 (10.9)	34.2 (9.8)	35.7 (10.4)	t = 1.32, p = 0.19^b^
Smoker [N] (males/females)	22 (18/4)	29 (19/10)	51 (37/14)	X^2^ = 2.28, p = 0.13^a^
Vocational qualification^d^ [score], median (IQR)	1 (1)	1 (1)	1 (1)	Z = 0.16, p = 0.87^c^
Duration of illness [years], mean (STD)		4.8 (5.6)		
Medication^e^		16 none		
		21 FGAs		
		7 SGAs		
PANSS [score], mean (STD)		85.7 (27.2)		
RT gain [ms], mean (STD)	272 (90)	370 (170)	321 (144)	t = 3.40, p = 0.001^b^
RT loss [ms], mean (STD)	273 (88)	375 (168)	324 (143)	t = 3.57, p = 0.001^b^
RT neutral [ms], mean (STD)	329 (105)	403 (157)	367 (139)	t = 2.54, p = 0.013^b^

Abbreviations: FGAs, first-generation antipsychotics; PANSS, Positive and Negative Syndrome Scale; RT, mean Reaction time across all cue conditions during MID task; SGAs, second-generation antipsychotics; STD, standard deviation.

^a^ By Pearsons's chi-square test.

^b^ By two-sample test.

^c^ By Mann–Whitney U test.

^d^ With pre-determined response options: (0) no professional qualification, (1) vocational training/apprenticeship, (2) advanced technical college, (3) university.

^e^ Mean Chlorpromazine Equivalent (CPE) = 405 ± 297 mg.

### Monetary Incentive Delay Task and Data Acquisition

Participants performed a monetary incentive delay task (MID task; [40,41]) during fMRI. The task invokes anticipation of reward and punishment. Depending on the performance in a simple reaction time task (button press) to a visual target a potential monetary gain, loss or no consequence is depicted at the end of the trial. Prior to fMRI acquisition, participants received information about the meaning of the cues. Participants were informed that they receive the earned money after completion of the scanning session. During the acquisition of the anatomical scan, participants practiced the task (without monetary payment). Each trial started with the presentation of a cue indicating whether subjects could win money, avoid losing money or obtain no money (neutral cue). The different magnitudes of the incentive (0.10 €; 0.60 € or 3 €) were indicated by the number of horizontal lines presented inside the cue image. Between cues and target, a variable delay was inserted. The application of an adaptive algorithm for target duration enabled subjects to succeed in about 67% of the trials. Successful trials were defined as button presses within the time frame of the target presentation. To control for neuronal artifacts due to motor response, participants were instructed to press the button as fast as possible regardless of the cue. A feedback display was presented after each trial to indicate the trial-related success. MR acquisition comprised anatomical and functional scans. The functional scans were splitted into two runs with altogether 144 trials consisting of 54 gain, 54 loss, and 36 neutral trials, which were presented in a random sequence (trial length 8 s, jittered mean intertrial interval 4 s; for a detailed description of the task, see Hägele and colleagues [31]).

### fMRI Data Acquisition

Images were acquired with a 1.5 T Magnetom VISION (Siemens) using a standard circularly polarized head coil (CP-Headcoil). Gradient-echo echo-planar imaging (GE-EPI, TR = 1.9 s, TE = 40 ms, flip angle = 90°, matrix = 64 × 64, voxel size = 4 mm × 4 mm × 3.3 mm) was used to produce eighteen slices approximately parallel to the bicommissural plane (ac-pc plane), covering the inferior part of the frontal lobe (superior border above the caudate nucleus), the entire temporal lobe, and large parts of the occipital region. fMRI volume acquisitions were time-locked to the offset of each cue and were thus acquired during anticipatory delay periods. Six fMRI volumes were acquired per trial, resulting in 450 volumes per run. High resolution anatomical images were acquired using a 3D MPRAGE sequence (Magnetization Prepared Rapid Gradient Echo, TR = 9.7 ms; TE = 4 ms; flip angle 12°; matrix = 256 × 256, voxel size 1 mm × 1 mm × 1 mm). A vacuum pad served to minimize head movements.

### fMRI Data Analysis

SPM8 (http://www.fil.ion.ucl.ac.uk/spm) was used for fMRI data analysis. To avoid non-steady state effects from T1 saturation the first three volumes of each functional time series were discarded. Volumes were realigned to the first volume to correct for between-scan movements and to remove signals correlated with head motion using sinc interpolation. Motion correction confirmed that no subjects showed more than 4 mm head movement during the run and less than 1 mm translation and 1° rotation in any dimension from one volume acquisition to the next. The anatomical image was coregistered to the mean functional image. The functional data set was coregistered with the anatomical volume based on the mean functional volume of the first run and spatially normalized to the standard MNI template using the algorithm implemented in SPM8 (12-parameter affine transformation followed by a non-linear warping using 7x8x7 harmonic basis functions to compensate anatomical distortions). Subsequently, the data were resampled to a resolution of 3 × 3 × 3 mm voxel size and smoothed using a 8 mm full-width half-maximum (FWHM) isotropic kernel. Functional MRI data were analyzed using the general linear model (GLM; [42]). Data analysis was performed by modelling the onsets of the three different conditions (cues for gain, loss and no monetary) as explanatory variables convolved with hemodynamic response function (gamma-variate function; [43]). Changes in the blood-oxygen level-dependent (BOLD) response were assessed using linear combinations of the estimated GLM parameters (betas) and are contained in the individual contrast images for the seven cue conditions, the target and the five feedback conditions (successful gain, non-successful gain, successful loss avoidance, non-successful loss-avoidance, neutral condition). Movement parameters derived from image realignment were included as additional regressors of no interest. For the anticipation phase the contrast image ‘gain vs. no outcome’ was computed combining the three different values for gain. Knutson and colleagues [41] suggested that neuronal activation during reward anticipation is stronger than during loss anticipation, and indeed, several studies observed small or non-significant differences in loss anticipation between healthy participants and patients with various disorders, and furthermore during the feedback phase [31,44,45]. To use a robust and strong contrast for the MVPA approach we therefore focused on reward anticipation in both fMRI and MVPA analyses. For standard univariate analysis, the individual contrast images entered a second-level random effects analysis to investigate the between-group differences with respect to the gain vs. no outcome contrast using a two-sample t-test (FDR corrected at q = 0.05, cluster level 30 voxels).

### Multivariate Pattern Analysis

MVPA was performed to investigate whether the clinical diagnosis can be determined on the basis of task-related regional activation patterns of the gain vs. no outcome contrast. Support vector machine classification (SVM; [46,47]) has been shown to be a powerful tool for statistical pattern analysis and proven to be a versatile and robust approach for analyzing functional neuroimaging data [21,48]. SVM is a binary classifier that finds the maximum margin separating hyperplane. Based on the training data the goal of SVM is to produce a model which predicts the target value y_i_ (label, diagnostic status) of the test instance i given only the test data attributes x_i_ (features, fMRI voxel).

Given a training set $S={\left\{(x_{i},y_{i})\right\}}_{i=1}^{m}$, where *x_i_* ∈ *R^d^* and *y_i_* ∈ {+1,−1} the standard form of the SVM objective with parameter C to scale the loss is

$$\underset{}{E_{C}(w)}=\frac{1}{2}{\left\|w\right\|}^{2}+C\sum_{i=1}^{n}l_{i}\left(\langle x_{i},w\rangle \right)$$

where w is the normal to the hyperplane and *l_i_*(*z*) denotes the loss function. The standard SVM objective is equivalent (proportional) to the following SVM objective,

$$\underset{}{E(w)}=\frac{\lambda }{2}{\left\|w\right\|}^{2}+\frac{1}{n}\sum_{i=1}^{n}l_{i}\left(\langle x_{i},w\rangle \right)\;\;if\;\lambda =\frac{1}{nC},\;\;C=\frac{1}{n\lambda }.$$

Using the hinge-loss function the following optimization problem has to be solved

$$\underset{w}{\arg\min}f(w)\;\;where\;f(w)=\frac{\lambda }{2}{\left\|w\right\|}^{2}+\frac{1}{n}\sum_{i=1}^{n}\max\{0,1-y_{i}\langle x_{i},w\rangle \}$$

while an ε-accurate solution $\hat{w}\text{ defined as }f(\hat{w})\leq f(w)+\varepsilon$ is quested by the applied optimization method. To solve the optimization problem in the primal space we used the Pegasos algorithm [49], a stochastic sub-gradient descent method (Matlab code is provided by Sebastien Paris, http://www.mathworks.com/matlabcentral/fileexchange/33621-fast-linear-binary-svm-classifier). While the traditional subgradient method uses the entire training set at each iteration step, Pegasos chooses randomly a single training example to estimate a sub-gradient of the objective, and a step with pre-determined step-size is taken in the opposite direction of the gradient. One of the advantages of the Pegasos algorithm lies in the fast final convergence of solving the optimization problem and the substitution of the cost parameter. The linear support vector classification (SVC) with Pegasos algorithm for solving the optimization problem was embedded in a searchlight approach to identify local brain patterns with informative signatures with respect to the clinical status [34,35]. For each voxel location within the scan volume, the data of the voxels within a searchlight sphere of six voxels in diameter were fed into the classifier with a leave-one-out cross-validation (LOOCV) scheme. For each cross-validation iteration data were partitioned into training and test sets, by excluding one different participant (n_test_ = 1), and the SVC classifier was trained on the data of the remaining participants (n_train_ = N-1, where N = 88). The trained classifier was then used to predict the label of the unseen test participant based on his/her data alone. This process was repeated leaving each participant out once to finally obtain an accuracy measure (percentage of correctly predicted labels) based on the number of correctly classified test samples. Note that the training set during each training iteration consisted of unequal sample sizes (43 and 44 datasets for each group, respectively). Although unequal group sizes during training can introduce a prediction bias towards the majority class, we decided to apply this procedure for the following reasons: (i) The impact of the bias can be regarded as neglible because the imbalance is small given the relatively large sample size; (ii) the direction of the bias is balanced across LOOCV because both groups contained equal sample sizes; (iii) to equalize sample sizes during training another participant from the other group has to be chosen based on an arbitrary selection scheme. Mapping this accuracy value into the center of the searchlight sphere and performing the LOOCV-searchlight procedure for all locations results in a brain map of decoding accuracies. Statistical significance of the overall classification accuracy was determined by permutation testing to generate empirical chance distributions of the accuracy measure for all decoded locations [50,51]. For this, the LOOCV procedure was repeated 10,000 times with a different random permutation of the training group labels. To maintain the spatial coherence, the permutation of the label was kept constant within each permutation step while in turn each permutation step comprised an entire LOOCV-searchlight decoding of all voxels within the scan volume. For each voxel the probability to receive the accuracy value for the actual labels by chance was estimated using the permutation-based histograms of chance accuracy values. In order to confine alpha inflation due to multiple comparisons we used the false discovery rate (FDR; [52]). FDR controls the average fraction of false positives (at q = 0.05) out of the set of all positive test results. As for the univariate case a minimum cluster size of 30 voxels was considered.

To directly compare the classification accuracies of the SVM analysis with its complement from the univariate approach, we generated accuracy maps for the univariate analysis by computing the Receiver-Operating Characteristic curve (ROC; [53]) for each brain voxel. The ROC curve displays the sensitivity versus 1-specificity at various discrimination thresholds and is used to determine the threshold with the best classification percentage over the available training set. In other words, for each voxel we estimated the optimal trade-off between misses and false positives which in turn represents the maximum reachable accuracy for correctly classifying the two groups when considering the univariate analysis. Accuracies were computed for the ventral striatum as our primary regions of interest. The ventral striatal region of interest was specified from the publication-based probabilistic Montreal Neurological Institute (MNI) atlas used as binary mask at the threshold of 0.75 probability (see http://hendrix.imm.dtu.dk/services/jerne/ninf/voi/index-alphabetic.html).

For the schizophrenia group, a linear support vector regression (SVR; [54]) was performed to test whether the severity of negative symptoms as measured with PANSS scale could be predicted from fMRI response patterns of the contrast 'monetary gain vs. no outcome' for voxels within the ventral striatal mask (for a review, see [55]). The cost parameter, which determines the influence of the misclassification on the objective function, was fixed at the default setting of C = 1. On the basis of previous findings [7] we hypothesized that voxels within the ventral striatum carry information especially on the severity of negative symptoms. For SVR we used a similar LOOCV-searchlight procedure as for the support vector classification: For each voxel of the ventral striatum, the data of the voxels within a searchlight sphere of four voxels in diameter were fed into the SVR with a leave-one-out cross-validation. Compared to SVC the searchlight sphere for SVR was smaller in diameter because the ventral striatal mask contained only 90 and 92 voxels for left and right ventral striatum, respectively. In each cross-validation step, the data were partitioned into training and test sets, by excluding a different schizophrenia patient, and the SVR was trained on the data of the other patients. The resulting regression model was then used to predict the PANSS score of the untrained patient based on his/her functional data alone. Conducting this LOOCV scheme for each patient yielded a vector with individual PANSS score estimates. Spearman correlation was used to examine the relation between PANSS score estimates and actual PANSS scores. The correlation coefficient was than mapped into the center of the searchlight sphere and the entire LOOCV-searchlight-SVR procedure was performed for all locations within the ventral striatal mask. Permutation tests were performed to obtain unbiased empirical chance distributions for the relationship of true and predicted PANSS scores within the ventral striatum. For this, the LOOCV-searchlight-SVR procedure described above was repeated 10,000 times, each time with a different random assignment of the true PANSS scores across patients in the training set ('null SVR models'). Analogous to the SVC approach, the permutation-based histogram of each voxel was used to estimate the probability to obtain the observed relationship between predicted and true PANSS scores by chance. FDR correction [52] was finally applied to curtail alpha inflation.

## Results

We examined the performance of the searchlight SVM classification on fMRI activation patterns in response to reward-indicating stimuli with the aim to decode the clinical status (schizophrenia patients vs. healthy control). For completeness, we also report the univariate group differences between healthy controls and schizophrenia patients for the contrast reward anticipation versus no gain.

### Behavioral Data

Mean reaction times (RT) are shown in Table 1. A mixed two-way ANOVA with the within-subject factor reward cue (neutral, gain, loss) and the between-subject factor group (control, patients) on RT showed a main effect of reward cue (F(1.26,105.63) = 48.32, p < 0.001) as reported previously [31]. There was also a main effect of group (F(1,84) = 9.89, p = 0.002), indicating shorter RT in the healthy control group (RT averaged across conditions 383 ms (STD 163 ms) in schizophrenia patients and 292 ms (STD 91 ms) in controls). There was a significant group × reward-cue interaction (F(1.26,105.63) = 4.25, p = 0.033). To further analyse the significant interaction separate one-way ANOVAS for the healthy control group and schizophrenia patients were conducted. For the healthy control group a significant main effect for the factor reward cue (F(1.19,48.58) = 34.08, p < 0.001) was revealed. Pairwise comparisons (Bonferroni corrected) showed that healthy controls responded significantly faster during gain vs. neutral trials (p < 0.001) and loss vs. neutral trials (p < 0.001). There was no significant difference between gain and loss trials (p = 1.0) The ANOVA for schizophrenia patients also revealed a significant main effect for the factor reward cue (F(1.36,58.28) = 14.74, p < 0.001). Pairwise comparisons showed that schizophrenia patients responded significantly faster during gain vs. neutral trials (p < 0.001) and loss vs. neutral trials (p = 0.001). There was no significant difference between gain and loss trials (p = 0.61). Thus the effect of reward and loss anticipation on RT revealed similar result patterns between groups, indicating that participants in both groups understood the paradigm and were engaged in the task to a similar extend. Please also note that the MID task was programmed to adjust to individual reaction times, so equal percentages of gains and losses for all participants were ensured [40,45].

### Univariate group differences in reward anticipation

**Fig 1:**
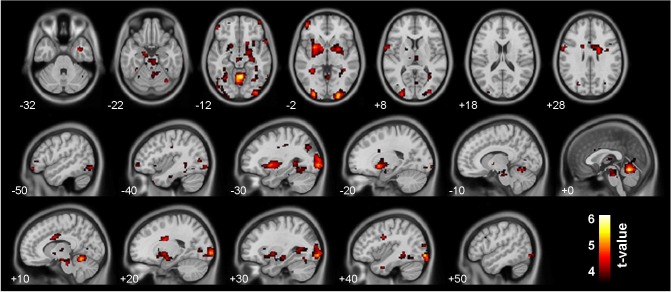
Group differences in reward anticipation. Results for the contrast reward anticipation versus no outcome for healthy controls > schizophrenia patients (thresholded at p < 0.05, FDR-corrected for multiple comparisons, cluster level 30 voxels). Healthy controls displayed significant larger activations in the ventral striatum, hippocampus, caudate body and substantia nigra during reward-indicating versus neutral cues.

As previously reported [31], BOLD activation during anticipation of monetary gain versus no gain was significantly reduced in schizophrenia patients compared to healthy subjects in the bilateral ventral striatum. As can be seen in Fig. 1, schizophrenia patients also showed reduced responses in the putamen, parahippocampal gyrus, cingulate gyrus, caudate, insula, amygdala and thalamus, as well as multiple frontal, temporal and occipital regions (Table 2).

**Table 2:** Activations for the contrast reward anticipation versus no outcome for healthy controls > schizophrenia patients.

			**MNI coordinates (mm)**		
**Cerebral region**	**Hem**	**t**	**x**	**y**	**z**
Middle frontal gyrus	L	4.75	−45	51	0
Precentral gyrus	L	3.74	−39	−15	33
	R	4.43	39	−3	30
Middle cingulate gyrus	R	4.44	6	3	30
Parahippocampal gyrus	R	4.52	15	−12	−15
Caudate body	L	4.22	−18	6	27
	R	4.58	21	−3	27
Putamen	L	5.09	−24	6	−6
Thalamus (dorsomedial NC)	R	4.14	3	−15	6
Amygdala	R	4.24	33	0	−33
Superior temporal gyrus	R	4.53	60	15	−15
Inferior temporal gyrus	R	4.07	63	−57	−15
Fusiform gyrus	L	4.26	−36	−42	−18
Precuneus	L	4.35	−27	−72	33
Cuneus	R	3.98	18	−75	3
Middle occipital gyrus	L	5.22	−30	−96	−3
Middle occipital gyrus	R	5.45	27	−96	−6
Brain stem	R	4.46	6	−27	−24
Vermis 4–5	R	6.16	6	−57	−15
Cerebellum-6	L	5.03	−6	−66	−15
	R	4.24	33	−69	−21

Abbreviations: Hem, Hemisphere; L, left; NC, nucleus; R, right.

### MVPA classification of schizophrenia patients and healthy controls

**Fig 2:**
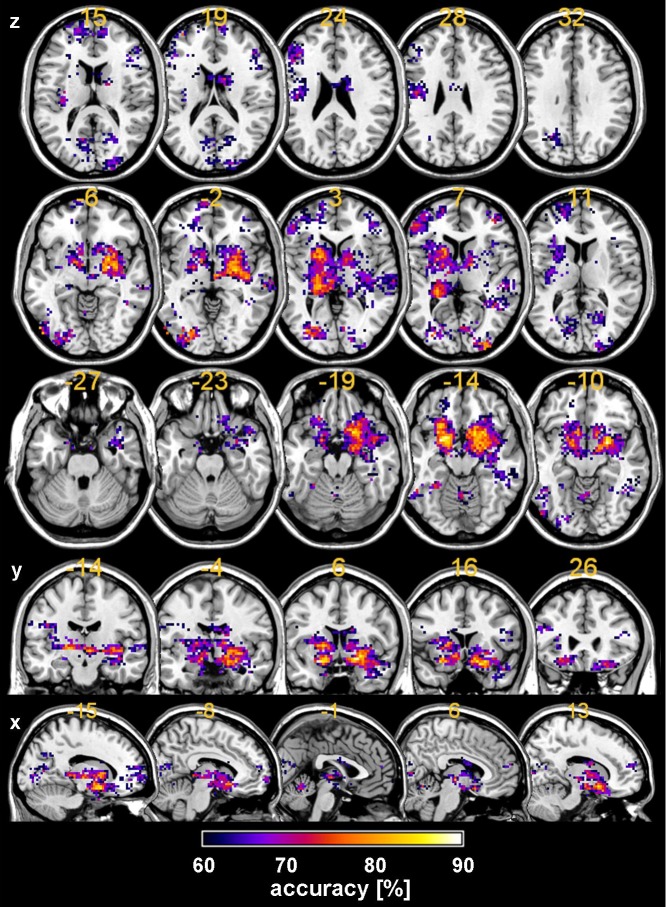
Brain areas that discriminated between schizophrenia patients and healthy control during reward anticipation using a multivariate classification approach. Accuracy scores (percent correct classification) from SVM searchlight decoding were colour-coded to display the classification performance. Letters x, y, z denote the axial, coronal and sagittal planes, respectively. The maps are thresholded at a significance level of p<0.05, FDR-corrected (cluster level 30 voxels).

Searchlight MVPA identified a distributed cortical network of frontal, temporal, occipital and midbrain regions with high classification accuracies. Across all voxels the mean of the lowest, medium and highest accuracies scores by chance, derived from the permutation-based random distributions, were 29.1%, 48.4%, and 66.5%, respectively. By contrast, maximal accuracy for the classification of patients vs. controls was obtained in the right pallidum ([MNI: 24, −6, −6], accuracy = 93%), bilateral putamen (left: [MNI: −24, 6, −15], accuracy = 90%; right: [MNI: 24, 3, −9], accuracy = 90%), right inferior frontal gyrus ([MNI: 18, 15, −18], accuracy = 86%), right nucleus accumbens ([MNI: 12, 12, −12], accuracy = 85%), right amygdala ([MNI: 27, −3, −15], accuracy = 83%), bilateral insula (left: [MNI: −27, 15, −15], accuracy = 84%; right: [MNI: 39, −18, −3], accuracy = 82%), bilateral thalamus (left: [MNI: −18, −24, 0], accuracy = 83%; right: [MNI: 6, −15, −3], accuracy = 82%), and left inferior temporal gyrus ([MNI: 19, −54, −75, −6], accuracy = 82%; p < 10^−5^ for all accuracies). Interestingly, for the twelve regions with the best accuracies (mean accuracy: 85%, range: 82–93%) the sensitivity (mean: 91%, range: 73–100%) was generally larger compared to the specificity score (mean: 79%, range: 64–93%). See Table 3 and Fig. 2 for further details.

**Table 3:** Multivariate classification of schizophrenia patients and healthy controls.

		**MNI coordinates (mm)**						**Accuracy within 6 mm sphere around peak voxel**		**Accuracy by chance at peak voxel**		
**Cerebral region**	**Hem**	**x**	**y**	**z**	**Accuracy**	**Sensitivity**	**Specificity**	**Min**	**Mean**	**Min**	**Mean**	Max
Medial frontal gyrus	R	12	57	15	**70.5**	88.6	52.3	60.2	64.3	30.7	48.7	65.9
Middle frontal gyrus	R	39	48	6	**75.0**	95.5	54.5	61.4	67.1	29.5	48.7	67.0
Inferior frontal gyrus	R	18	15	−18	**86.4**	95.5	77.3	62.5	75.7	29.5	48.4	67.0
Inferior frontal gyrus	R	42	42	0	**70.5**	84.1	56.8	61.4	65.6	30.7	48.6	68.2
Pallidum	L	−18	6	3	**84.1**	93.2	75.0	62.5	74.4	27.3	48.3	65.9
	R	24	−6	−6	**93.2**	97.7	88.6	60.2	75.1	30.7	48.5	65.9
Putamen	L	−24	6	−15	**89.8**	86.4	93.2	60.2	73.1	30.7	48.2	65.9
	R	24	3	−9	**89.8**	100.0	79.5	70.5	79.1	28.4	48.3	67.0
Thalamus	L	−18	−24	0	**83.0**	100.0	65.9	60.2	73.9	30.7	48.2	68.2
	R	6	−15	−3	**81.8**	81.8	81.8	60.2	68.6	29.5	48.3	64.8
Insula	L	−27	15	−15	**84.1**	95.5	72.7	61.4	72.8	29.5	48.3	67.0
	R	39	−18	−3	**81.8**	88.6	75.0	61.4	71.9	30.7	48.2	69.3
Amygdala	R	27	−3	−15	**83.0**	88.6	77.3	62.5	73.0	29.5	48.2	64.8
Middle temporal gyrus	L	−48	−69	−6	**70.5**	95.5	45.5	60.2	63.4	28.4	48.1	69.3
Inferior temporal gyrus	L	−54	−75	−6	**81.8**	100.0	63.6	60.2	68.5	26.1	48.2	64.8
Fusiform gyrus	L	−30	−48	−12	**79.5**	70.5	88.6	61.4	68.9	27.3	48.5	63.6
Precuneus	L	−12	−66	15	**72.7**	95.5	50.0	60.2	63.6	30.7	48.6	69.3
Cuneus	L	−15	−84	3	**69.3**	97.7	40.9	61.4	65.8	29.5	48.5	68.2
Middle occipital gyrus	L	−33	−81	0	**81.8**	88.6	75.0	63.6	72.0	30.7	48.6	65.9
	R	24	−93	9	**81.8**	93.2	70.5	61.4	67.2	29.5	48.5	64.8
Lingual gyrus	L	−21	−78	−3	**80.7**	79.5	81.8	61.4	69.0	30.7	48.7	67.0
Vermis 4–5	L	−3	−60	−12	**71.6**	81.8	61.4	61.4	67.7	29.5	48.1	64.8
	R	6	−60	−12	**73.9**	81.8	65.9	62.5	66.6	29.5	48.1	64.8

Abbreviations: Hem, Hemisphere; L, left; Max, Maximum; Min, Minimum; R, right.

Accuracy, sensitivity and specificity of peak cluster maxima.

### Comparison of univariate and multivariate classification of patients and healthy controls for the ventral striatum

**Fig 3:**
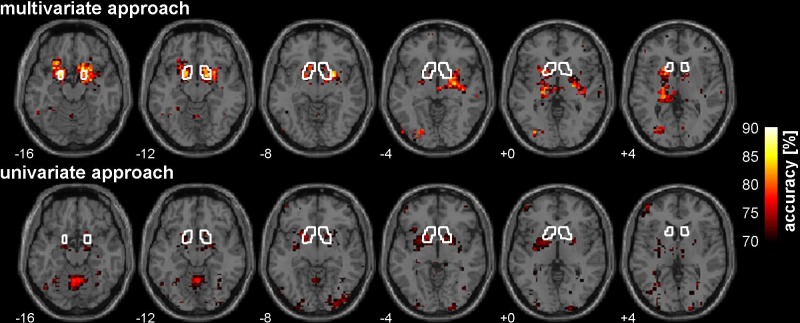
Classification performance comparison. The top and bottom panels depict percent correct classification rates (accuracies) obtained from the multivariate (linear SVM) and univariate (ROC) classification approach, respectively. The white line denotes the mask boundary of the ventral striatum. For illustrative reasons the accuracies where thresholded at 70% thus fewer significant voxels are displayed in the figure compared to the actually survived number of voxels after FDR correction.

For our primary region of interest, the ventral striatum, we compared the accuracies of the univariate approach derived from Receiver-Operating Characteristic curve analysis (ROC; [53]) with those of MVPA using the SVM classifier. For comparability reasons, FDR correction was applied for both approaches. As expected, larger maximum accuracy scores were observed in the multivariate case for both the left and the right mask of the ventral striatum (left: [MNI: −18, 2, −11], accuracy = 87%; right: [MNI: 18, 5, −11], accuracy = 88%) when compared to the univariate approach (left: [MNI: −18, 8, −5], accuracy = 75%; right: [MNI: 15, 11, −8], accuracy = 73%). Within the ventral striatum sensitivity and specificity of the peak voxel for the two approaches were compared using McNemar's test. The marginal proportions were significantly different from each other (X^2^ = 5.88, p = 0.015), indicating that MVPA provides a better prediction performance compared to the univariate model. With the multivariate approach a substantially greater number of voxels survived FDR correction within the mask of the ventral striatum compared to the univariate analysis (percent significant voxels with the ventral striatum mask: multivariate: L = 91%, R = 77%; univariate: L = 56%, R = 65%). See Table 4 and Fig. 3 for further details.

**Table 4:** Comparison of univariate and multivariate classification performance for the ventral striatum.

	**Univariate approach**			**Multivariate approach**		
	**L & R**	**L**	**R**	**L & R**	**L**	**R**
**Max accuracy**	75.0	75.0	72.7	87.5	86.4	87.5
MNI xyz	−18 8–5	−18 8–5	15 11–8	18 5–11	−18 2–11	18 5–11
**Mean accuracy (STD)** *	66.5 (5.1)	66.7 (5.8)	66.3 (4.4)	71.4 (7.2)	71.5 (6.9)	71.4 (7.5)
Median accuracy*	69.3	71	69.3	70.5	70.5	69.3
Min accuracy*	63.6	65.9	63.6	60.2	60.2	61.4
# significant voxel*	110	50	60	153	82	71
% significant voxel*	60.4	55.6	65.2	84.1	91.1	77.2

Using a ventral striatal mask with 182 voxels (90 and 92 voxels for left and right ventral striatum, respectively).

Abbreviations: L, left; Min, minimum; Max, maximum; R, right; STD, standard deviation.

*Parameter refers to the voxels within the ventral striatal mask.

### Multivariate prediction of the PANSS negative scale

**Fig 4:**
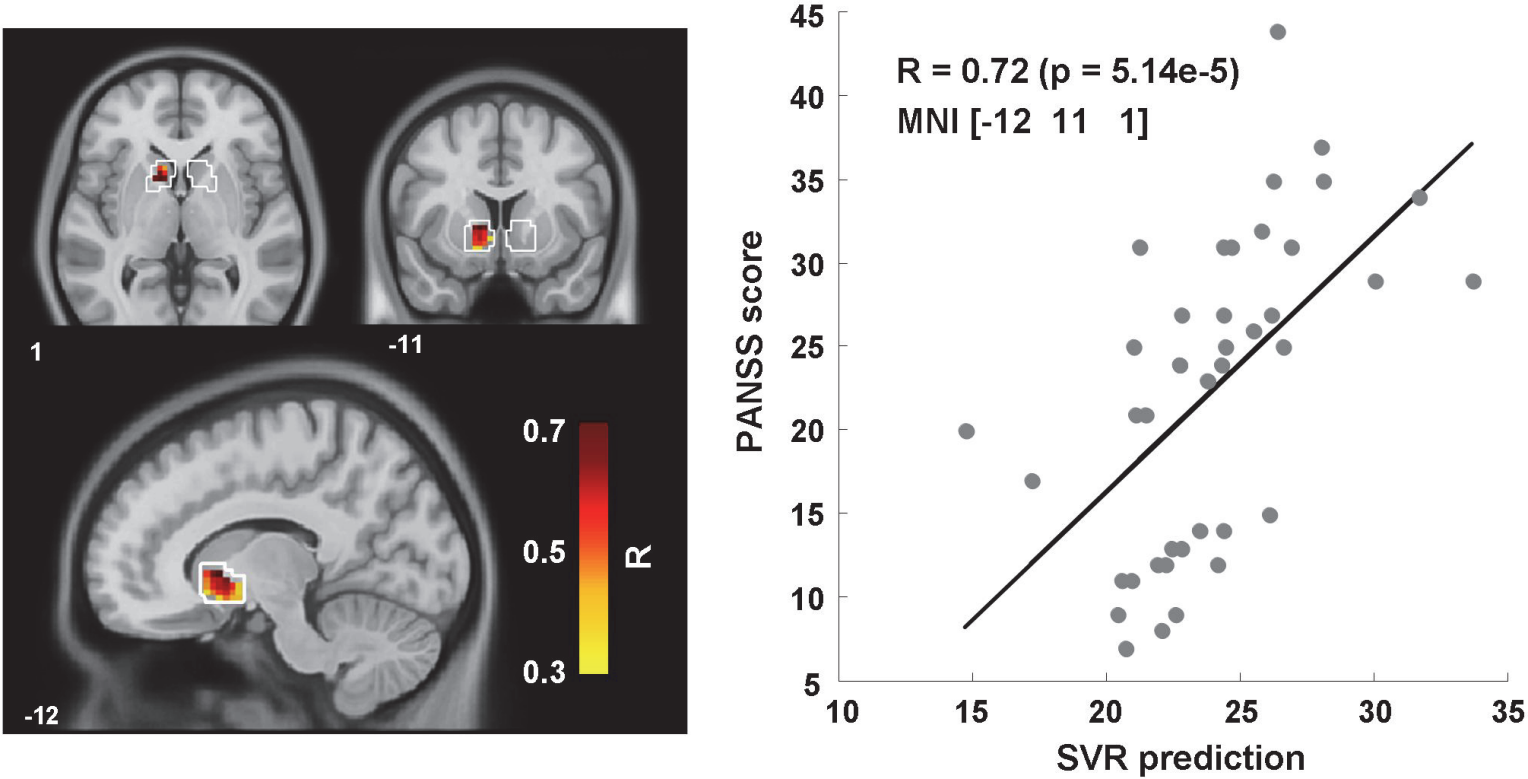
Support vector regression (SVR) with PANSS negative scale for the schizophrenia group. For the fMRI contrast monetary gain vs. no outcome there was a tight relationship between PANSS negative symptom scores and those predicted with SVR from activation patterns left ventral striatum within the clinical group. The right panel shows the correlation for the voxel (MNI: −12, 11, 1) within the left ventral striatum with the strongest relationship (R = 0.72) between actual and predicted PANSS negative scores. Each dot represents a schizophrenia patient.

Leave-one-out SVR (LIBSVM; [54]) was used to investigate the relationship of gain anticipation and the PANSS negative scale in schizophrenia patients within the ventral striatum. The ventral striatum was chosen because previous univariate analyses revealed an inverse relationship between ventral striatal activation and the severity of negative symptoms [7,10]. The severity of negative symptoms as measured with PANSS could be predicted from the left ventral striatal activation pattern in response to monetary gain vs. no outcome: Within the left ventral striatum support vector regression revealed the strongest relationship with the PANSS negative scale for the searchlight sphere with the center coordinate at MNI = [−12, 11, 1] and a Spearman correlation coefficient of R = 0.72 (p = 5.14e-5; see Fig. 4). For the same center coordinate, predictions by chance revealed a minimum, mean and maximum correlation coefficient of R = −0.83, R = −0.154 and R = 0.66, respectively. Across all voxels within the ventral striatal mask the mean of the lowest, medium and highest coefficients by chance were −0.85, −0.15, and 0.62, respectively. Note that negative coefficients represent no predictive information regarding the function estimation between PANSS scores and predicted PANSS values from multiple voxel data using SVR.

Altogether, the tight relationship between the actual PANSS negative scores and those predicted by SVR indicates that voxels within the ventral striatum carry information with respect to the severity of negative symptoms in schizophrenia patients.

## Discussion

In this study we used searchlight MVPA of regional fMRI activation patterns in response to anticipation of monetary reward for diagnostic classification of schizophrenia patients vs. healthy control participants. Regional activation patterns with the highest accuracy scores for the discrimination between schizophrenia patients and controls were observed in subcortical regions such as the pallidum, putamen, nucleus accumbens, as well as in the inferior frontal gyrus and insular cortex. In line with previous reports, the univariate comparison of the groups revealed a reduced BOLD activation to reward anticipation in the ventral striatum [6–9,36] and a distributed network of regions in schizophrenia patients compared to healthy controls [7,11–14]. For the left and right ventral striatum the multivariate classification revealed one of the highest class prediction rates, which where found to be larger compared to those computed on the basis of ROC-curves from univariate analysis. The lower accuracies of the univariate approach can be attributed to the fact that the mass-univariate analysis treats each voxel independently and therefore does not take into account information that reflects task-related group differences in neural activity that are spatially distributed. Conversely, the searchlight SVM incorporates redundant but also additive information from spatially correlated neighbouring voxels, thereby improving class prediction [33].

In line with previous studies, both the univariate comparison and the multivariate classification of the two groups show that the ventral striatum, a key region in reward processing and encoding of the incentive salience of rewarding stimuli [56–59], exhibits differential activation for schizophrenia patients compared to healthy controls. Previous studies have found an inverse relationship between the severity of negative symptoms and the magnitude of BOLD activation in the ventral striatum during reward anticipation [7,11,60,61,44]. Our current results corroborate the notion that the negative symptoms of schizophrenia are related to ventral striatal activation. They go beyond these previous reports by now showing a significant correlation of actual PANSS negative ratings with those predicted by support vector regression, thus indicating that not only the magnitude of the ventral striatal responses but also the activation pattern in this region is informative with regard to psychopathology. Our study thus provides additional evidence that reward-associated neural activity in the left ventral striatum is coupled to the severity of negative symptoms in schizophrenia patients and supports the hypothesis that reduced motivation or anhedonia is linked with ventral striatal dysfunction [62,63].

Importantly, we used a multivariate searchlight approach [32,33] to investigate which brain regions contain activation patterns with valuable diagnostic information for the discrimination of schizophrenia patients and healthy controls. This approach successfully exposed distinct brain regions that have been observed in previous univariate studies [6–9,11–14,31,36]. Our results therefore confirm the significance of these regions in the pathophysiology of schizophrenia and highlight the usefulness of MVPA searchlight analysis for the identification of regional activation patterns that can help the diagnostic classification of clinical groups.

Note that in general specificity was somewhat smaller than sensitivity. We attribute this to the differences in the variance between groups. While both groups group showed the same frequency of voxels with violations against the normal distribution (Shapiro-Wilk test: 8.7% and 7.5% of the voxels, respectively), the variance within the schizophrenia group was smaller compared to healthy controls: Although Levene's Test for Homogeneity of Variances rejected on average only 7.1% of the voxels, numerically, 80.2% of these voxels showed a larger variance within healthy controls compared to schizophrenia patients. We conducted SVC simulations with normally distributed random data and systematically varied the variance, skewness and kurtosis parameters in one of the groups. The results of these simulations support the observation that the trade-off between sensitivity and specificity is determined by group differences in the variance parameter rather changes in skewness and kurtosis. Accordingly, the larger variance in the healthy control group may have led to consistently larger sensitivity and smaller specificity scores.

Because the multivariate searchlight combines signals from several voxels within a region, it is more sensitive to local information and shows a larger classification performance compared to univariate analysis, but at the same time provides regionally specific information. The simplicity of implementation and interpretability of regional pattern as well as the avoidance of critical prerequisites of whole brain decoding such as the choice of feature selection or dimensionality reduction technique and optimal feature size emerged as pivotal advantages of the searchlight technique compared to whole brain decoding strategies [64,65]. Apart from advantages of SVR and SVC machines some points have to be taken into account: The choice of the kernel function, the optimal selection of the meta-parameters (weighting of misclassifications, C and size of the insensitive loss region, ε) and the kernel parameters have an impact on the generalization performance and raise the problem of empirical tuning. While a nonlinear kernel provides equal or better prediction performances, the parameters of the solved model are difficult to interpret. Finally, the support vector regression as used here yields regression estimations without providing the direction of the relationship between predicted values based on multivariate data (voxel values) and the predictor variable (severity of negative symptoms).

Our results not only show that MVPA improves classification accuracy when compared to univariate methods but also suggest that the searchlight-based analysis of local pattern information can yield classification accuracies that may be even useful for individualized clinical decisions [66]. The used multivariate approach can be seen as proof of concept for the attempt to bridge the gap between the univariate approach, which merely depicts regional differences, and the diagnostic classification of the individual based on multivariate pattern information. The goal of this approach is not to replace clinical diagnosis. However, with the advance of machine learning techniques, MVPA has the potential to identify neuroimaging–based patterns with pathophysiological relevance and may serve as a basis for improved classification and differential diagnosis in the future.

Taken together, in this proof of concept study we were able to identify neurobiological markers of high diagnostic information for schizophrenia using searchlight MVPA. Our results show that searchlight MVPA can be used to substantially increase the accuracy of diagnostic classification on the basis of task-related fMRI signal patterns in a regionally specific way. This approach might help to identify biological diagnostic markers for schizophrenia that could be integrated in diagnostic systems in the future.
